# Inchinkoto, the Traditional Japanese Kampo Medicine, Enhances Intestinal Epithelial Barrier Function In Vitro

**DOI:** 10.1155/2022/4139812

**Published:** 2022-09-29

**Authors:** Ayaka Nakao, Ailing Hu, Takuji Yamaguchi, Masahiro Tabuchi, Yasushi Ikarashi, Hiroyuki Kobayashi

**Affiliations:** ^1^Department of Personalized Kampo Medicine, Juntendo University Graduate School of Medicine, Bunkyo-ku, Tokyo 113-8421, Japan; ^2^Department of Hospital Administration, Juntendo University Graduate School of Medicine, Bunkyo-ku, Tokyo 113-8421, Japan

## Abstract

Inchinkoto (ICKT), a traditional herbal medicine that is often used as a hepatoprotective drug in Japan, has pharmacological properties that include antioxidant, anti-inflammatory, and choleretic actions. Genipin is a metabolite of geniposide and the most abundant ingredient of ICKT; furthermore, it is considered to be the active substance responsible for its pharmacological properties in the liver. Drugs with such pharmacological characteristics are expected to prevent intestinal barrier dysfunction, which causes inflammatory bowel diseases (IBDs). However, no studies have investigated the effects of ICKT on the intestinal epithelial barrier. Therefore, we investigated the activity of ICKT in intestinal tight junctions by using cultured Caco-2 cell monolayers. The action of the compound on tight junctions was examined by measuring transepithelial electrical resistance (TEER) and sodium fluorescein (Na-F) permeability in the presence or absence of lipopolysaccharide (LPS). Moreover, the expression of the tight junction protein claudin-1 was assessed by using immunofluorescent staining. ICKT and genipin increased TEER and decreased Na-F permeability, which was suggestive of enhanced intestinal epithelial barrier function. Moreover, they prevented the LPS-induced destruction of the barrier, i.e., a decrease in TEER and an increase in Na-F permeability. Immunofluorescence staining revealed a high claudin-1 expression level on the cell surface, whereas exposure to LPS downregulated claudin-1. In turn, ICKT and genipin prevented the LPS-mediated reduction of claudin-1. These results suggest that ICKT enhances intestinal epithelial barrier function by upregulating claudin-1. Furthermore, genipin contributed to these effects. ICKT may be a promising medicine for the prevention and treatment of diseases associated with intestinal barrier disruption, such as IBD, obesity, and metabolic disorders.

## 1. Introduction

Adjacent intestinal epithelia form tight junctions, which are essential for the function of the physical intestinal barrier. These junctions are made up of the transmembrane proteins occludin and claudin, which reside between the intestinal epithelium cells and cytosolic scaffold zonula occludens proteins [1]. Tight junctions composed of such proteins bring epithelial cells in close contact with one another and prevent the entrance of inflammatory substances derived from foods and intestinal bacteria into the body [2, 3]. The loss of barrier integrity contributes to inflammatory bowel disease (IBD), obesity, and metabolic disorders [4].

Dysfunction of this barrier is caused by various pathological, toxicological, and physical factors, including oxidative stress, inflammation, and decreased bile secretion [4, 5], e.g., the oxidative stress caused by reactive oxygen and nitrogen species produced by various stresses, such as exercise-induced ischemia, high-fat and high-carbohydrate diet-induced nutritional stress, and injuries caused by ischemia/reperfusion [6]. Oxidative stress disrupts the intestinal barrier (tight junctions). Cystine, a substrate of the antioxidant glutathione, improves oxidative stress-induced barrier dysfunction by enhancing glutathione synthesis [6]. The endotoxin lipopolysaccharide (LPS), which is a component of the outer wall of Gram-negative bacteria, promotes the production of proinflammatory mediators, such as interleukin-6 (IL-6) and tumor necrosis factor-*α* (TNF-*α*), which subsequently cause barrier dysfunction [7]. Paeoniflorin, which has anti-inflammatory properties, is a bioactive compound from *Paeonia lactiflora* Pallas plants that prevents LPS-induced intestinal barrier disruption [8]. The fucoidan dietary substance is a sulfated fucose-enriched polysaccharide that is found in the extracellular matrix of brown algae and has anti-inflammatory and antioxidant effects, thereby improving hydrogen peroxide-induced intestinal barrier dysfunction [9]. In addition, the decrease in bile secretion into the intestine caused by biliary atresia and cirrhosis reduces the intestinal intraluminal concentrations of bile acids, thus causing intestinal inflammation and barrier destruction, which in turn induces bacterial translocation [10, 11].

The series of adverse effects caused by bile deficiency is attenuated by the oral administration of bile acids [12]. The findings mentioned above collectively suggest that antioxidant, anti-inflammatory, and bile-promoting (or choleretic) ingredients or drugs maintain and strengthen the integrity and function of the intestinal barrier. This has implications for the management of intestinal diseases, such as IBD.

The human colon carcinoma cell line Caco-2 has been widely used in vitro to investigate intestinal epithelial functions and pathologies because of the similarity between these cells and human enterocyte structure and functions [6, 8, 9]. In fact, most of the evidence on the function of the intestinal barrier and tight junctions stems from studies that used Caco-2 cell monolayer models.

The orally administered inchinkoto (ICKT) traditional Japanese herbal medicine (called Kampo in Japan) comprises the following compounds: *Artemisia capillaris* Flos, Gardeniae Fructus, and Rhei Rhizoma. The Japanese Ministry of Health, Labour, and Welfare has approved ICKT for treating the following conditions: jaundice, cirrhosis, nephrotic syndrome, urticaria, and mouth ulcer. Among these indications, ICKT is often used as a choleretic and hepatoprotective drug in patients with liver diseases, including jaundice [13–15]. Recent basic and clinical studies have demonstrated that the antioxidant, anti-inflammatory, and choleretic actions of ICKT are responsible for its hepatoprotective effects; oral ICKT has been reported to prevent liver damage in rats by dampening the exacerbated inflammatory responses and oxidative stress caused by the combined effects of ischemia-reperfusion and subsequent hepatectomy [16]. Moreover, ICKT inhibits nitric oxide (NO) production, which causes liver damage, by blocking the induction of NO synthase gene expression by IL-1*β* in primary cultured rat hepatocytes [17, 18]. ICKT has also been demonstrated to increase bile flow and the biliary excretion of bilirubin conjugates in rats [19, 20] and humans [13, 14, 21]. These effects are associated with an enhanced choleretic activity mediated by the multidrug resistance-associated protein 2 and an antioxidative action mediated by a nuclear factor E2-related factor-dependent mechanism [19–21].

Plants are important sources of prophylactic and therapeutic bioactive molecules and are expected to contribute to traditional and modern medicine as essential regimens [22, 23]. Plants and crude drugs have been reported to contain many bioactive ingredients, namely, phenolic and polyphenolic compounds (such as flavonoids and essential oil components), which have antioxidant and anti-inflammatory effects [23–25]. Thus, the wide range of protective functions, such as antioxidant, anti-inflammatory, and neuroprotective effects, of natural products and their derivatives in various plants have been reported to be useful in the treatment of diabetes, depression [26], stress, anxiety [27], and Alzheimer's disease [22, 28]. Similarly, ICKT contains several bioactive substances, including geniposide and the major ingredients of *Artemisia capillaris* Flos (6,7-dimethylesculetin and capillarisin). These ingredients are thought to be involved in the pharmacological properties of ICKT, such as its antioxidant, anti-inflammatory, and choleretic actions [20, 21, 29]. In particular, it has been reported that genipin, which is a metabolite of geniposide and the most abundant ingredient of ICKT, is closely involved in the hepatoprotective action of ICKT [20, 29]. Ingested geniposide is converted to genipin by intestinal bacteria via bacterial *β*-glucosidase action [16, 20]. Genipin is then absorbed by the intestinal mucosa and transported to the liver via portal circulation [13, 14].

These findings led to the inference that ICKT, in addition to its choleretic, anti-inflammatory, and antioxidant effects, may also be involved in the maintenance and strengthening of intestinal barrier function. This further led to the speculation that ICKT may prevent and treat diseases associated with intestinal barrier disruption, such as IBD, obesity, and metabolic disorders. In addition, biliary atresia and IBD have been reported to disrupt the intestinal barrier and promote the development of hepatitis, cirrhosis, and liver cancer [30, 31]. If ICKT strengthens the intestinal barrier and protects it from barrier disruption, it could also lead to the prevention and treatment of liver disease caused by such intestinal disorders and provide a new mechanism to support the hepatoprotective effects of ICKT. In addition, genipin is thought to be a potent active ingredient in the hepatoprotective effects of ICKT [20, 29]; however, although ICKT showed beneficial effects on the intestinal barrier, it is unclear whether genipin is the active ingredient in the barrier effects. To the best of our knowledge, the therapeutic effects of ICKT on the intestinal barrier have not been investigated.

Therefore, in this study, to clarify whether ICKT acts on the intestinal barrier, the effects of ICKT and genipin on intact Caco-2 monolayers and LPS-induced dysfunction barriers were examined. Barrier function was investigated by measuring transepithelial electrical resistance (TEER), the paracellular permeability of sodium fluorescein (Na-F), and tight junction protein expression by using immunofluorescent staining.

## 2. Materials and Methods

### 2.1. Test Substances and Reagents

**Figure 1:**
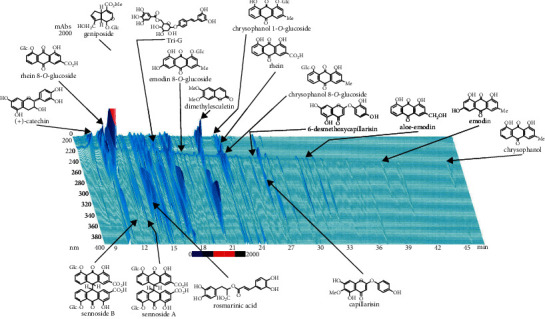
Three-dimensional high-performance liquid chromatogram with ultraviolet detection of the inchinkoto methanol extract.

The ICKT used in this experiment was a dry powdered extract (lot no. M05021) supplied by Tsumura & Co. (Tokyo, Japan) comprising a mixture of *Artemisia capillaris* Flos, Gardeniae Fructus, and Rhei Rhizoma with a weight ratio of 4 : 3 : 1. We purchased genipin (lot no. KPX4H-CJ) from Tokyo Chemical Industry Co., Ltd. (Tokyo, Japan).  Figure 1 shows the three-dimensional chromatogram of the ICKT extract. In this context, the dried extract of ICKT (1 g) was dissolved in 20 mL of methanol under ultrasonication for 30 min, followed by centrifugation at 3,000 rpm for 5 min. Subsequently, the supernatant was filtrated through a 0.45 *μ*m membrane filter. Next, a sample of the filtrate (30 *μ*L) was injected into a high-performance liquid chromatography (HPLC) instrument equipped with ultraviolet detection. This led to the identification of at least 17 ingredients in the methanol extract of ICKT.

LPS and Na-F were purchased from Sigma-Aldrich, Co., Ltd. (Saint Louis, MO, USA), the mouse anti-human claudin-1 ALexa Fluor 488-conjugated antibody was purchased from Zymed Laboratories (San Francisco, CA, USA), and other chemicals were purchased from commercial sources.

### 2.2. Preparation of Compound Solutions

The ICKT and genipin solutions were prepared in dimethyl sulfoxide (DMSO), with the final concentration of DMSO adjusted to 0.5% in each experiment.

### 2.3. Cell Culture

Caco-2 cells purchased from Life Technologies Corporation (Grand, NY, USA) were cultured according to a previously reported procedure [32]. Briefly, the cells were incubated in Dulbecco's modified Eagle's medium (DMEM) supplemented with 10% fetal bovine serum, 1% nonessential amino acids, 100 U/mL penicillin, 100 *µ*g/mL streptomycin, and 292 *µ*g/mL L-glutamine in a CO_2_ incubator (Life Technologies Corporation) under atmospheric conditions of 37°C and 5% CO_2_. The cells were cultured until they reached 80%–90% confluence, after which the medium was changed every 2 days. Confluent cells were used in the in vitro experiments.

### 2.4. Examination of the Effects of the Test Substances on Cell Viability

The effects of ICKT and genipin on Caco-2 cell viability were evaluated with tetrazolium salt sodium 3'-(1-[(phenylamino)-carbonyl]-3,4-tetrazolium)-bis(4-methoxy-6-nitro)benzene-sulfonic acid hydrate (XTT) by using a colorimetric assay kit (XTT Cell Proliferation Kit®; Roche Diagnostics, Mannheim, Germany) [33]. Cell viability was measured according to the manufacturer's protocol. Briefly, Caco-2 cells (1.0 × 10^4^ cells/well) were seeded onto 96-well plates and cultured for 24 h in supplemented DMEM. This was followed by medium replacement with DMEM, including various concentrations of ICKT (50, 100, and 200 *μ*g/mL), genipin (10 *μ*g/mL), or vehicle, and incubation for 24, 48, or 72 h. Subsequently, the medium was replaced with 50 *µ*L of XTT solution, and the cells were incubated for an additional 2 h at 37°C in a CO_2_ incubator, followed by the measurement of cell viability at a wavelength of 450 nm by using a microplate reader (Thermo Fisher Scientific, Tokyo, Japan). Cell viability was determined as a percentage of the absorbance of the compound-treated cells relative to that of the vehicle-treated control cells (100% viability).

### 2.5. Examination of Caco-2 Cell Tight Junction Integrity

**Figure 2:**
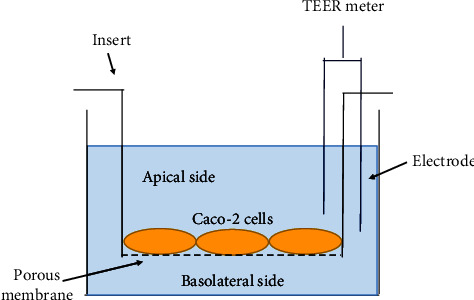
Measurement of transepithelial electrical resistance (TEER). The Transwell was partitioned into apical and basolateral compartments by inserting an insert. Caco-2 cells (4 ×  10^5^ cells/well) were seeded onto the upper surface of the insert membrane (0.4 *µ*m pore size) and cultured in a differentiation medium. TEER was measured by using an EVOM3 epithelial volt-ohm TEER meter equipped with an STX3 “chopstick” electrode.

According to a previously reported procedure, cultures of Caco-2 cell monolayers for tight junction formation were performed by using 12-well Transwell plates (Corning Life Sciences, Acton, MA, USA) [32]. First, Caco-2 cells (4 × 10^5^ cells/well) were seeded onto the upper surface of the well membrane insert (pore size, 0.4 *µ*m) and cultured in intestinal epithelial differentiation medium (Oriental Yeast Co., Ltd., Tokyo, Japan) for 10 days in a CO_2_ incubator at 37°C to confirm tight junction integrity during culture. The medium was changed every 2 days. TEER, which reflects cell-to-cell tightness [34], was measured daily by using an EVOM3 epithelial voltage resistance meter (World Precision Instruments, Sarasota, Florida, USA) equipped with the STX2-plus “chopstick” electrode shown in Figure 2.

### 2.6. Examination of the Effects of the Test Substances on Tight Junction Function

#### 2.6.1. Direct Effects of ICKT and Genipin on the Caco-2 Monolayer Barrier

The direct effects of ICKT and genipin on the epithelial cell barrier were assessed by using Caco-2 cell monolayers cultured in 12-well Transwell plates for 10 days according to the procedure described in subsection 2.5. The treatments were performed by replacing the apical medium with a medium containing ICKT (50, 100, and 200 *μ*g/mL), genipin (10 *μ*g/mL), or vehicle (control), followed by incubation at 37°C for 24 h in a CO_2_ incubator and TEER measurement. In this experiment, a genipin concentration of 10 *μ*g/mL was chosen with reference to previous reports that showed the effectiveness of this concentration of the compound in cell culture experiments [19].

For further verification of tight junction integrity, Na-F permeability across the Caco-2 cell monolayer was measured as reported previously [35]. Briefly, after measuring TEER, the apical medium in the insert was replaced by 0.5 mL of medium containing 10 *µ*g/mL Na-F. After 1 h of incubation, the Na-F concentration in the basolateral medium was measured by using an Infinite M200 fluorescence multiwell plate reader (TECAN, Männedorf, Switzerland; excitation wavelength: 485 nm; emission wavelength: 535 nm).

#### 2.6.2. Effects of ICKT and Genipin on the LPS-Induced Destruction of the Caco-2 Cell Monolayer Barrier

LPS-induced destruction of the Caco-2 cell monolayer barrier was carried out as described previously [34]. The apical medium of Caco-2 cell monolayers cultured for 10 days in 12-well Transwell plates was replaced with a fresh medium containing LSP (100 *µ*g/mL), LSP + test substances (ICKT: 50, 100, and 200 *μ*g/mL; or genipin: 10 *μ*g/mL), or a medium without these compounds (as a control). Subsequently, the plate was incubated at 37°C for 24 h in a CO_2_ incubator, and TEER and Na-F permeability were measured by using the procedure described in subsection 2.6.1.

#### 2.6.3. Immunofluorescence Staining of Claudin-1

Immunofluorescence staining of the tight junction protein claudin-1 was performed according to a procedure employed previously to investigate the effect of the test material on LPS-induced Caco-2 cell monolayer barrier disruption [9]. Briefly, after TEER measurement, the cells were washed twice with cold phosphate-buffered saline (PBS) and fixed with a 10% buffered neutral formalin solution for 10 min. Then, the cells from the porous membrane of the insert of the Transwell were mounted onto slides, followed by overnight incubation with a mouse anti-human claudin-1 antibody at 4°C. Next, the cells were washed with PBS and incubated with an ALexa Fluor 488-conjugated antibody, followed by a final wash. Immunofluorescence was examined and imaged using a fluorescence microscope (Nikon Eclipse 80i; Tokyo, Japan).

### 2.7. Statistical Analysis

All data are presented as the mean ± standard error of the mean. One-way analysis of variance (ANOVA) and post hoc Dunnett's test were used to determine the significance of associations. The threshold of significance was set at *P* < 0.05.

## 3. Results

### 3.1. Cell Viability

Caco-2 cell viability after exposure to ICKT (50, 100, and 200 *μ*g/mL) and genipin (10 *μ*g/mL) was measured by using the XTT assay. The viable cell rate was >90% in the concentration range of both test substances, indicating that the ICKT and genipin concentrations used in subsequent experiments were almost nontoxic to Caco-2 cells (data not shown).

### 3.2. Relationship between Days in Culture and Caco-2 Cell Tight Junction Formation

**Figure 3:**
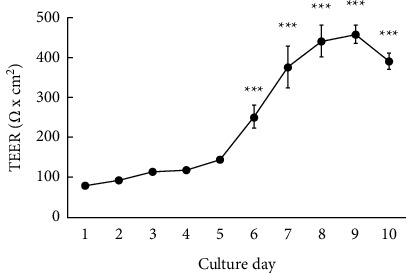
Relationship between tight junction formation and days in the culture of Caco-2 cells. Transepithelial electrical resistance was measured daily for 10 days. Each value represents the mean ± standard error of the mean (*n* = 6). ^*∗∗∗*^*P* < 0.001 vs. day 1: statistical significance was assessed by using Dunnett's test following the one-way analysis of variance.

Figure 3 shows the state of the development of tight junctions relative to the days in culture as assessed by measuring TEER. One-way ANOVA detected significant differences in the culture-day factor (*F*_9,50_ = 40.00, *P* < 0.001), whereas the post hoc analysis showed that the TEER values increased rapidly as culture progressed from day 6 to day 10 compared with day 1, which was set as the control (*P* < 0.001). The increased TEER levels reached a plateau between day 8 and day 10, indicating that tight junctions were well formed. Therefore, cells with a tight junction with TEER values >350 Ω × cm^2^ were used in subsequent experiments.

### 3.3. Direct Effects of ICKT and Genipin on the Caco-2 Monolayer Barrier

**Figure 4:**
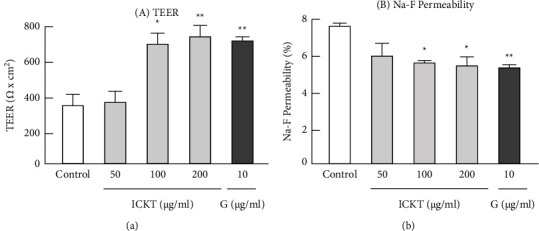
Direct effects of inchinkoto and genipin on the Caco-2 monolayer barrier. Each figure shows transepithelial electrical resistance (a) and Na-F permeability (b). Each value represents the mean ± standard error of the mean (*n* = 6). ^*∗*^*P* < 0.05 and ^*∗∗*^*P* < 0.01 vs. control: statistical significance was assessed by using Dunnett's test following the one-way analysis of variance.

Figure 4 shows the direct effects of ICKT and genipin on the tight junction integrity markers TEER Figure 4(a) and Na-F permeability Figure 4(b) in Caco-2 cell monolayers. One-way ANOVA detected significant differences in both TEER (*F*_4,25_ = 8.028, *P* < 0.001) and Na-F permeability (F_4,25_ = 6.720, *P* < 0.01). The post hoc analysis showed that exposure to ICKT (*P* < 0.05 at 100 *µ*g/mL and *P* < 0.01 at 200 *µ*g/mL) and genipin (*P* < 0.01 at 10 *µ*g/mL) resulted in a significant increase in the TEER values compared with the control; this was accompanied by a significant decrease in Na-F permeability (*P* < 0.05 at 100 and 200 *µ*g/mL ICKT; and *P* < 0.01 at 10 *µ*g/mL genipin). These findings strongly suggest that the test compounds enhance both the integrity and the function of the intestinal epithelial barrier.

### 3.4. Effects of ICKT and Genipin on the LPS-Induced Destruction of the Caco-2 Cell Monolayer Barrier

**Figure 5:**
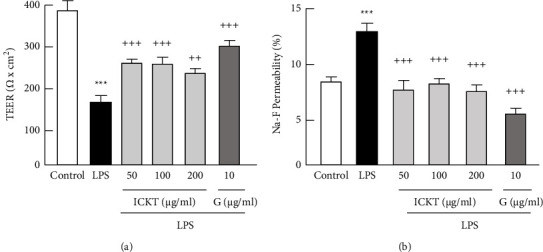
Preventive effects of inchinkoto and genipin against the 100 *µ*g/mL lipopolysaccharide (LPS)-induced destruction of the Caco-2 cell monolayer barrier. Each figure shows transepithelial electrical resistance (a) and Na-F permeability (b). Each value represents the mean ± standard error of the mean (*n* = 6). ^*∗∗∗*^*P* < 0.001 vs. the control, and ^††^*P* < 0.01 and ^†††^*P* < 0.001 vs. LPS. Statistical significance was assessed by using Dunnett's test following a one-way analysis of variance.

Figure 5 shows the results obtained for TEER Figure 5(a) and Na-F permeability Figure 5(b), which were measured to assess the preventive effects of ICKT and genipin on the LPS-induced Caco-2 monolayer barrier destruction. One-way ANOVA detected significant differences in both TEER (*F*_5,30_ = 14.02, *P* < 0.001) and Na-F permeability (*F*_5,30_ = 35.67, *P* < 0.001). The post hoc analysis revealed that LPS (100 *µ*g/mL) treatment significantly decreased TEER (*P* < 0.001) and increased Na-F permeability (*P* < 0.001) in the cell monolayers, indicating that LPS destroyed the Caco-2 monolayer barrier. Furthermore, both ICKT and genipin significantly inhibited the LPS-induced decrease in TEER (*P* < 0.01 – 0.001 at 50–200 *µ*g/mL ICKT; and *P* < 0.001 at 10 *µ*g/mL genipin) and increased Na-F permeability (*P* < 0.001 at 50–200 *µ*g/mL ICKT; and *P* < 0.001 at 10 *µ*g/mL genipin). These results indicate that ICKT and genipin prevented the LPS-induced destruction of the Caco-2 monolayer barrier.

### 3.5. Immunofluorescence Staining to Determine Claudin-1 Expression in Response to Exposure to the Test Compounds

**Figure 6:**
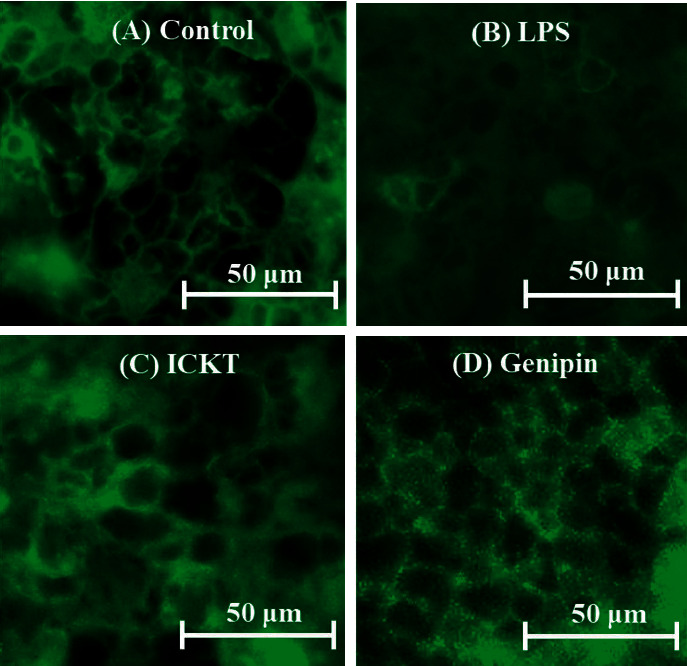
Preventive effects of inchinkoto (ICKT) and genipin against lipopolysaccharide (LPS)-induced disruption of the tight junction protein claudin-1. Claudin-1 was detected by using immunofluorescence staining. Images show the control (a), 100 *µ*g/mL LPS (b), LPS + 200 *μ*g/mL ICKT (c), and LPS + 10 *μ*g/mL genipin (d).

To investigate the activity of the test compounds on the expression of the tight junction protein claudin-1, we performed immunofluorescence staining on cell monolayers that were incubated for 24 h in a control medium, LPS (100 *µ*g/mL), LPS + ICKT (200 *μ*g/mL), or LPS + genipin (10 *μ*g/mL). As shown in Figure 6, the control cells exhibited a high expression level of claudin-1 Figure 6(a) and LPS-induced disruption of claudin-1 integrity Figure 6(b), whereas ICKT Figure 6(c) and genipin Figure 6(d) prevented the LPS-induced perturbation of claudin-1 expression.

## 4. Discussion

Accumulating evidence suggests that plant-derived components or drugs with antioxidant, anti-inflammatory or biliary-promoting effects protect against the intestinal barrier disruption induced by various diseases, including IBD, obesity, and metabolic disorders [4, 6–11]. Therefore, we speculated that ICKT, which has anti-inflammatory, antioxidant, and bile-secretion-promoting activities [16–21], can effectively strengthen and protect the intestinal epithelial barrier. However, ICKT has been studied only for its hepatoprotective effects [13–21]; to the best of our knowledge, its effects on the intestinal barrier have not been investigated. As predicted, by using cultured Caco-2 cells, this study reported for the first time that ICKT had barrier-enhancing and protective effects and that, among its ingredients, at least genipin was involved in the observed effects.

Here, we measured TEER and Na-F paracellular permeability as indicators of barrier function integrity. The TEER values increase when the barrier of the cultured cell layer is formed or strengthened and decrease when it is weakened or destroyed. In contrast, the permeability of small molecules between cells (i.e., paracellular permeability) decreases when the barrier is formed or is strengthened and increases when the barrier is weakened or destroyed [6, 8, 9, 35]. However, the Caco-2 cell monolayer tight junction integrity is affected by culture conditions, such as the cell source, number of seeded cells, and duration of culture [36]. Therefore, before evaluating the effects of the test substance on barrier function, we first examined the tight junction integrity during culture under our experimental conditions by measuring TEER. Subsequently, we confirmed that the TEER values increased with days in culture, reaching a stable level after 8 days (Figure 3). This result suggested that a cell culture period of at least 8 days is required to complete the formation of tight junctions under our experimental conditions. Most studies using Caco-2 cells have applied monolayers with TEER values >250–300 Ω × cm^2^, which are believed to indicate tight junction integrity [32, 34, 37]. Therefore, we used cells cultured for 10 days with TEER values >350 Ω ×  cm^2^ for experimentation.

Exposure to ICKT and genipin significantly increased the TEER value of cell monolayers with tight junction integrity compared with the control, which was also supported by a significant decrease in Na-F permeability (Figure 4). This result suggests that both substances directly enhance the intestinal epithelial barrier function, although the underlying mechanisms remained unclear in this experiment. The intercellular junctions (or cell-cell junctions) formed between adjacent cells involve the tight junction proteins occludin and claudin, as well as the cytosolic scaffolding protein zonal occludens and junctional adhesion molecules [1, 38]. Therefore, the enhancement of the intestinal epithelial barrier function via the effects of ICKT and genipin may be directly modulated by the expression of intercellular junction-related proteins. To elucidate the detailed mechanism underlying the enhancing effects of ICKT and genipin on intestinal epithelial barrier function, their direct effects on the expression of intercellular junction-related proteins should be further studied and verified by using quantitative polymerase chain reaction and Western blotting.

Next, we investigated whether the two test substances protect against the LPS-induced disruption of the Caco-2 cell monolayer barrier. LPS induces barrier dysfunction by activating proinflammatory cytokines and oxidative stress through the Toll-like receptor 4 [7, 8, 25, 34, 39]. Moreover, it has been observed that claudin-1/4 and occludin are downregulated in the intestinal epithelia of patients with ulcerative colitis [40]. In this study, LPS decreased TEER levels and increased Na-F permeability. These changes were inhibited by cotreatment with ICKT or genipin (Figure 5). In addition, the expression of claudin-1 in Caco-2 monolayers with tight junction integrity was disrupted by exposure to LPS; in turn, this effect was prevented by cotreatment with ICKT or genipin (Figure 6). These results suggest that both substances protect against the tight junction destruction induced by LPS. ICKT and genipin have been shown to have antioxidant and anti-inflammatory properties [16–18]. Therefore, the protective effects of these two substances against LPS-induced barrier or tight junction disruption are thought to occur via antioxidant and anti-inflammatory mechanisms, in addition to their previously described barrier-enhancing effects. Considering that the loss of intestinal barrier integrity contributes to IBD, obesity, and metabolic disorders [4], the barrier-enhancing and protective effects of ICKT may efficiently treat these diseases, in addition to their previously reported hepatoprotective efficacies against jaundice and cirrhosis. Nevertheless, to clarify whether the results of this in vitro study translate into in vivo effects, further studies are necessary to verify their efficacy in animal models with intestinal barrier disorders.

Genipin is an intestinal bacterial metabolite of geniposide, which is the main component of Gardeniae Fructus, the most abundant ingredient of ICKT [18, 19]. Orally ingested geniposide is converted to genipin by *β*-glucosidase of the intestinal bacteria, absorbed by the intestinal mucosa, and transported to the liver via the portosystemic circulation [13, 14, 38]. Genipin is considered to be one of the components involved in the hepatoprotective action of ICKT through its antioxidant, anti-inflammatory, and bile secretion-promoting actions [20, 29]. However, whether genipin is the main active ingredient triggering the intestinal barrier-enhancing effect of ICKT and its preventive effects against LPS-induced barrier destruction remains unclear. In this study, as described previously, the effects of genipin mimicked the beneficial effects of ICKT, suggesting that genipin plays an important role in the effects of ICKT against intestinal barrier functions. In this regard, we also found that genipin was the main active ingredient triggering this effect.

However, the involvement of components other than genipin in this effect cannot be ruled out. It has been widely shown that several plant-derived compounds, such as phenolic, polyphenolic, and essential oil components, exert significant anti-inflammatory effects [23, 25, 41]. A group of these anti-inflammatory compounds has been classified as cytokine-suppressive anti-inflammatory drugs, which target the proinflammatory activator protein 1 and nuclear factor-*κ*B signaling pathways and inhibit the expression of many proinflammatory cytokines, such as IL-1, IL-6, TNF-*α*, and NO [41, 42]. ICKT, which is a hepatoprotective drug for various liver diseases that is composed of three crude drugs, contains abundant bioactive substances in addition to genipin [13, 16, 19]. For example, the three-dimensional HPLC analysis of the ICKT extract shown in Figure 1 detected 17 ingredients, including phenols, polyphenols, flavonoids, and geniposide. Moreover, it has been demonstrated that ICKT attenuates inflammatory responses and oxidative stress (gene expression level of inducible NO synthase) in the rat liver following ischemia-reperfusion and subsequent hepatectomy [16]. Similar effects were observed after the administration of genipin. However, the effects of the latter were not as potent as those afforded by ICKT [16], suggesting that the combination of the multiple components of ICKT is necessary for the full expression of its beneficial effects. Generally, traditional herbal medicines are a mixture of several crude drugs that consist of many ingredients, which may systemically control a complex network system formed by numerous biomolecules [43]. The administration of such medicines is in accordance with a previous theory of multicomponent drugs [44]. The additive and/or synergistic effects of multiple components may be associated with the pharmacological actions of natural plants, including Kampo medicine [45]. Future investigations of ICKT are necessary for this context.

Biliary atresia and IBD have been reported to disrupt the intestinal barrier and promote the development of hepatitis, cirrhosis, and liver cancer [30, 31]. Strengthening of the intestinal barrier and protection against barrier disruption may lead to the prevention and treatment of such liver diseases. Therefore, the strengthening and preventive effects of ICKT on tight junctions should be considered novel mechanisms that support the hepatoprotective effects of ICKT.

## 5. Conclusions

We used a Caco-2 monolayer barrier model to demonstrate that ICKT and genipin enhanced normal intestinal epithelial barrier function and they prevented the LPS-induced disruption of intestinal epithelial barrier function. These results suggest that ICKT has enhancing and protective effects on intestinal epithelial barrier function and that genipin is one of the active ingredients responsible for those effects. However, the in vitro design of the study has inherent limitations. In the future, it is necessary to investigate drug activity against intestinal inflammation in vivo by using animal models [46], such as mice with dextran sulfate sodium-induced colitis.

## Data Availability

The data used to support the findings of this study are available from the corresponding author upon request.
